# Rapid white matter changes in children with conduct problems during a parenting intervention

**DOI:** 10.1038/s41398-023-02635-8

**Published:** 2023-11-04

**Authors:** Suzanne O’ Brien, Arjun Sethi, James Blair, Essi Viding, Ahmad Beyh, Mitul A. Mehta, Robert Dallyn, Christine Ecker, Marija M. Petrinovic, Moira Doolan, Nigel Blackwood, Marco Catani, Declan G. M. Murphy, Stephen Scott, Michael C. Craig

**Affiliations:** 1 Department of Forensic and Neurodevelopmental Sciences, Institute of Psychiatry, Psychology and Neuroscience, King’s College London, London, UK; 2 Research Unit at Child and Adolescent Mental Health Center Copenhagen, Capital Region of Denmark, Copenhagen, Denmark; 3 Institute of Clinical Medicine, University of Copenhagen, Copenhagen, Denmark; 4 Division of Psychology and Language Sciences, University College London, London, UK; 5 Department of Neuroimaging, Institute of Psychiatry, Psychology and Neuroscience, King’s College London, London, UK; 6 Department of Child and Adolescent Psychiatry, University Hospital of the Goethe University, Frankfurt am Main, Germany; 7 Department of Child and Adolescent Psychiatry, Institute of Psychiatry, Psychology and Neuroscience, King’s College London, London, UK; 8 NatBrainLab IRCCS Syllab, SDN, Naples, Italy; 9 National Female Hormone Clinic, Maudsley Hospital, London, UK

**Keywords:** Predictive markers, Neuroscience

## Abstract

Studies report that the microstructural integrity of the uncinate fasciculus (UF; connecting the anterior temporal lobe to the orbitofrontal cortex) is abnormal in adults with psychopathy and children with conduct problems (CP), especially those with high callous-unemotional (CU) traits. However, it is unknown if these abnormalities are *‘fixed’* or *‘reversible’*. Therefore, we tested the hypothesis that a reduction in CP symptoms, following a parenting intervention, would be associated with altered microstructural integrity in the UF. Using diffusion tensor imaging tractography we studied microstructural differences (mean diffusivity (MD) and radial diffusivity (RD)) in the UF of 43 typically developing (TD) and 67 boys with CP before and after a 14-week parenting intervention. We also assessed whether clinical response in CP symptoms or CU traits explained changes in microstructure following the intervention. Prior to intervention, measures of MD and RD in the UF were increased in CP compared to TD boys. Following intervention, we found that the CP group had a significant reduction in RD and MD. Further, these microstructural changes were driven by the group of children whose CU traits improved (but not CP symptoms as hypothesized). No significant microstructural changes were observed in the TD group. Our findings suggest, for the first time, that microstructural abnormalities in the brains of children with CP may be reversible following parenting intervention.

## Introduction

Conduct Problems (CP) are characterised by repetitive and persistent antisocial behaviour (ASB) and are one of the most common paediatric disorders in children [1]. Children with severe CP have a 5–10-fold risk of mental illness, substance abuse, criminality, unemployment, and early death in comparison to non-CP youth [2, 3]. CP not only poses a significant burden on the affected individual and their victims, but also on society and the economy, with evidence showing that children who exhibit life-course persistent CP account for a greater service burden than their peers across criminal justice, healthcare, and social service sectors in adulthood [4]. The risk of life-course persistent CP is greatest in children with ‘early-onset’ CP (i.e., onset before 10 years old) [5] and higher levels of callous-unemotional (CU) traits [6].

Currently, the most effective treatment to reduce this risk involves early intervention with group parenting programs [7–11], but around 50% of children do not respond to treatment [12]. This is likely due to the heterogeneity of CP, which is probably underpinned by biological differences in CP subtypes. For instance, previous research has fractionated CP into different subtypes typically based on ‘age of onset’ (i.e., childhood-onset compared to adolescent-onset CP) [13] and the presence or absence of CU traits [14]. However, to date, studies have not identified if specific biological differences (a) can predict treatment response, or (b) can change in children with CP whose antisocial behaviour improves (‘CP improvers’) or persists (‘CP persisters’).

One of the more robust biological findings associated with CP severity involves the uncinate fasciculus (UF), a white matter tract connecting the temporal lobe with the insular and orbitofrontal cortex [15, 16] and the subgenual cingulate cortex [17]. Whilst studies have not suggested that ASB is specific to this tract, accumulating evidence has indicated that brain regions which connect the UF, including the amygdala, the subgenual cingulate cortex and the frontopolar and ventromedial prefrontal cortex (and the structural and functional connections between these regions) may play a critical role in antisocial and psychopathic behaviours [18–24]. Microstructural abnormalities in this tract, relative to typically developing (TD) controls, have been reported in children with disruptive behaviours [25], adolescents with CP [26–28], and adults with psychopathy [29–31]. For instance, recent studies have reported reduced markers of white matter organization (i.e., reduced fractional anisotropy (FA)) in adults with psychopathy and children with disruptive behaviours [25, 29–32], whilst in adolescents with CP these markers appear to be increased compared to their TD peers [26–28]. However, to date, no studies have reported whether these differences remain fixed, or change, following improvement in antisocial behaviour.

Therefore, in the current study (which was an observational pre-post design), we analysed UF microstructure in a group of children with CP (before and after their parents had completed a parenting program) in comparison to a TD control group (at two equivalent time points). We hypothesised that:

- UF abnormalities in children with CP, compared to TD controls, will reduce following a parenting program (i.e., a group-by-time effect driven by the CP group).
- Reduction in UF abnormalities will be greatest in ‘CP improvers’, compared to ‘CP persisters’ or TD controls (i.e., a group-by-time interaction driven by the ‘CP improvers’ group).

## Methods

### Sample

We recruited 5–10 year old boys with CP (*n* = 67), and their parents, whilst on a wait-list to receive a 14-week parenting program. Boys with CP were recruited from two parenting programmes in the UK (the Incredible Years (IY) and Triple P) [33, 34]. Each programme required the child’s main caregiver to attend facilitated, weekly group sessions which focussed on various components (such as play, praise, rewards, limit setting, consequences, timeout), and parents/caregivers completed ‘homework’ between meetings. Families were referred to parenting groups from Child and Adolescent Mental Health Services (CAMHS), Local Authorities and Social Enterprises and attended weekly group training sessions. We also recruited age-matched TD control boys (*n* = 43), from the same inner-London schools and geographical areas. Initial inclusion criteria to the CP group required a score of ≥3 on CP scale of the Strengths and Difficulties Questionnaire (SDQ) [35]. Exclusion criteria in both groups included a clinical diagnosis of ASD, neurological abnormality or a full-scale IQ < 80. Boys with CP underwent behavioural and diffusion tensor imaging (DTI) analysis before (T1), and after (T2) their parents completed the parenting program (17.75 ± 5.3 weeks from T1 assessment). These assessments were replicated in TD boys at the same time points, albeit their parents did not participate in a parenting program. The sample size in the current study was chosen based on previous DTI studies (with smaller sample sizes) which have reported robust differences in white matter microstructure between children with CP and TD [36].

Written consent was obtained from all participants and ethical approval was granted by NRES Committee London-Westminster (IRAS Project ID:170367, REC Reference Number:15/LO/0696).

### Assessments and research diagnosis

CP symptoms were assessed at each time point using the Parental Account of Children’s Symptoms (PACS) as the primary outcome measure. This semi-structured clinical interview uses specific investigator-based criteria to assess both the frequency and severity of antisocial behaviours (e.g., aggression, destruction of property, disobedience etc.) and is highly predictive of later psychosocial outcomes [37]. The PACS scores eight categories of disruptive behaviour (telling lies; stealing; temper tantrums; rudeness; disobedience; refusal to go to bed; destructiveness; aggressiveness) on the ‘level of severity’ of each category of behaviour (0–3) and the frequency of that category (*‘Never or less than weekly’*; *‘1–2 days a week’*; *‘3–6 days a week’*; *‘Daily’*). To discern a clinically meaningful level of symptom reduction, we applied a minimally important clinical difference (MICD) approach [38, 39]. This used a pre-defined cut-off of 0.4 standard deviations (SD) from baseline PACS score, across the entire clinical cohort. This cut-off was based on 0.6 SD being associated with maximum user satisfaction (~92%) [40].

At both time points children’s behaviour was further assessed using the parent-rated SDQ, Inventory of Callous-Unemotional Traits (ICU) [41] and Conners-3 ADHD assessment [42]. Further, at baseline only, parents completed the Social Communications Questionnaire (SCQ) and maternal education was documented. Boys also completed the Wechsler Abbreviated Scale of Intelligence (WASI) [43] and a handedness questionnaire [44].

### Image acquisition

All participants underwent MRI scanning at each timepoint at the Centre for Neuroimaging Sciences, King’s College London (KCL). Diffusion-MRI data were acquired using a 3 T (GE Healthcare MR750) MRI scanner with a 32-channel receive-only RF head-coil.

Diffusion-weighted images (DWI) were acquired with a spin-echo echo planar imaging pulse sequence with the following parameters: FOV = 256×256 mm^2^; voxel size = 2x2x2 mm^3^; TE = 70 ms; TR = 12 s; 60 diffusion gradient directions; b-value = 1500 s/mm^2^; 6 non-diffusion-weighted (B0) volumes. In addition, 6 B0 volumes were acquired using the opposite phase encoding direction for susceptibility distortion correction. All data underwent a full quality control check, where all B0 and diffusion weighted volumes were visually inspected for motion artefacts, image corruption and signal drop-out effects. Subjects (at individual timepoints) were excluded if head motion parameters were two or more SD’s away from the mean (of all participants), or if a subject had ≥10 DWIs or ≥4 B0’s removed due to bad quality data.

### Diffusion MRI data processing

DWI data were denoised [44] and corrected for Gibb’s ringing artefacts [45] using TORTOISE [46, 47]. An off-resonance field was estimated in *topup* using the pairs of B0 images acquired with opposite phase encoding directions [48]. Simultaneous correction for motion, eddy current distortions, and susceptibility distortions (using the *topup* field) was then performed in *eddy* [49] with outlier slice replacement [50] and slice-to-volume motion correction [51].Tractography based on the tensor model and the Euler tracking algorithm [52] was performed in StarTrack (https://www.mr-startrack.com/) according to the following parameters: FA threshold = 0.20; step size = 1 mm; angle threshold: 35°. Tensor-derived maps, including fractional anisotropy (FA), mean diffusivity (MD), axial diffusivity (AD), and radial diffusivity (RD) were calculated. Anisotropic power (AP) maps [53], which are diffusion-derived maps that contain a good contrast in both grey and white matter, were also computed at this stage.

**Fig. 1:**
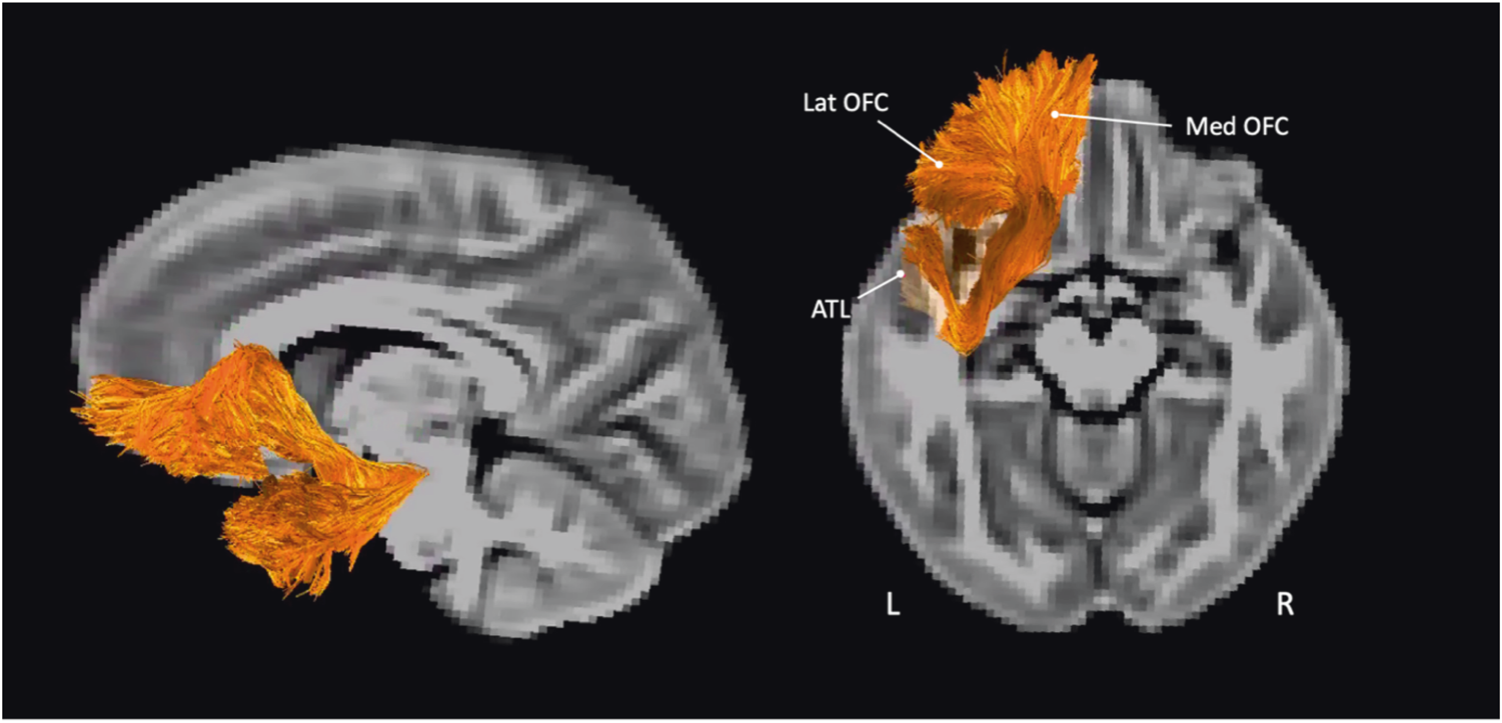
Tractography representation of the left uncinate fasciculus which extends from the anterior temporal lobe (ATL) towards the medial (Med) and lateral (Lat) orbitofrontal cortex (OFC). Tractography representation is from the MegaTrack dataset overlayed on a group anisotropic power (AP) map, representing 110 individuals across two timepoints.

MegaTrack [54], a semi-automatic group dissection approach, was adopted to account for the large number of subjects. For this purpose, each subject’s AP maps of time points 1 and 2 were used to create a subject-level template which is not biased toward either time point. The subject-specific AP templates were then combined to create a group-level AP template. All image normalisation steps were performed using Advanced Normalization Tools (ANTs) [55]. The resulting transformations were applied to each subject’s native space tractogram, then all tractograms were concatenated to create the final ‘mega’ tractogram for virtual dissection. Virtual dissections were performed in TrackVis (http://trackvis.org/). Reconstruction of the UF (Fig. 1) was performed one hemisphere at a time using a two region of interest (ROI) approach [56] using sphere ROIs. The first inclusion ROI was defined in the anterior temporal lobe and the second ROI was placed around the white matter of the anterior floor of the external/extreme capsule. Additional exclusion ROIs were used to manually remove any streamlines that did not belong to the UF. Once virtual dissections were completed, the MegaTrack framework was used to extract tract-specific measurements from each subject’s native space data. These included macrostructural metrics (track count and volume) and microstructural metrics (FA, MD, RD, and AD).

### Statistical analysis

Clinical variables between the control group and the CP group were analysed in IBM SPSS version 27.0, using linear mixed models. To assess whether the CP group overall responded to the intervention, we ran the analysis on the CP group only with Time (pre-intervention & post-intervention) as fixed effects with a random subjects factor. We then divided the CP group into ‘improvers’ and ‘persisters’ using the MCID approach as described above and analysed differences between these two groups using linear mixed effects models with Group (Improvers, Persisters), and Time (pre-intervention & post-intervention) as fixed effects with a random subjects factor. For the neuroimaging data, linear mixed effects models were also used to compare differences in FA, MD, RD and AD in each of the tracts of interest. Linear mixed effects models were run for all DTI measures (FA, MA, RD, MD) with Group (Controls, CP/ Controls, Improvers, Persisters), Time (pre-intervention & post-intervention) and Hemisphere (left, right) as fixed effects, and a random subjects factor. Significant *Time-by-Group (and Time-by-Group-by-Hemisphere)* effects were examined to assess for any microstructural changes over time between the groups. All analyses controlled for age, IQ, ADHD, maternal education (i.e., a measure of socio-economic status (SES)) and head motion in scanner. Reported *p*-values for AD and RD measures were adjusted for the false discovery rate (FDR) using the Benjamini-Hochberg procedure at *q* = 0.05 [57]. As MD and FA are combined measures of parallel (AD) and perpendicular (RD) diffusivity [58], we employed FDR correction on the two independent measures (AD and RD) only, as correcting for outcome measures which are interdependent is overly conservative, increasing Type II error risk [59]. As a result of the findings that emerged from our second hypothesis (see Results section), further *post hoc* analyses were conducted. As CU traits and ADHD also decreased in the CP group post-treatment, we used the same MICD approach as described earlier, whereby ‘improvers’ and ‘persisters’ were respectively defined by the presence or absence of a 0.4 SD reduction in ADHD or CU traits. To determine which grouping method best described our findings (i.e. was it change in CP, CU traits or ADHD), a likelihood-ratio test was performed in RStudio (2020) version 1.3.1093 (using R programming language version 3.6.3) to compare goodness of fit between our models.

## Results

### Demographics

The CP and TD control groups were matched on age and length of time between scans. There were no significant differences between groups for handedness, motion in scanner or days between scanning sessions. Differences in ADHD between the groups were observed, as well as differences in SES and IQ (Table 1). Individuals whose CP improved during the intervention (CP-improvers) and those whose CP persisted (CP-persisters) also differed from TD controls on ADHD, IQ and SES (Table 2). Four children from the CP group had missing PACS data for at least one of the timepoints and therefore it could not be assessed if these boys were CP improvers or persisters. Hence, these four subjects were excluded from the analysis examining CP symptom change (Hypothesis 2).

**Table 1:** Sample Characteristics for the Conduct Problem group and Control Group.

		Mean (SD)	*F*	*p*
	Controls	CP		
*n*	43	67		
Age (months)	101.80 (18.35)	103.12(18.10)	0.132	*p* = 0.717
IQ	109.16 (15.5)	102.72 (13.72)	5.13	*p* = 0.025
Handedness	−6.72 (3.68)	−5.92 (6.07)	0.36	*p* = 0.548
SES (Maternal Education)	5.68 (2.33)	4.18 (2.65)	8.45	*p* = 0.005
ADHD	16.10 (9.79)	53.94 (16.26)	186.93	*p* < 0.01
Motion in Scanner	0.36 (0.29)	0.44 (0.33)	1.93	*p* = 0.167
CP (PACS) T1	0.63 (0.36)	1.55 (0.43)	132.12	*p* < 0.001
CP (PACS) T2	0.57 (0.35)	1.37 (0.46)	89.58	*p* < 0.001
CP (SDQ) T1	1.04 (1.16)	5.48 (2.15)	149.05	*p* < 0.001
CP (SDQ) T2	0.90 (0.83)	4.35 (2.38)	82.61	*p* < 0.001
CU traits T1	15.34 (6.62)	34.14 (11.35)	96.04	*p* < 0.001
CU traits T2	15.89 (7.67)	30.82 (12.29)	50.30	*p* < 0.001
Days between T1 & T2 scans	122.62 (27.63)	124.31 (37.02)	0.06	*p* = 0.798

*CP* Conduct Problems, *IQ* Intelligence Quotient, SES socio-economic status, *ADHD* attention deficit hyperactivity disorder, *T1* Timepoint 1, *T2* Timepoint 2, *SD* standard deviation, *PACS* parental account of children’s symptoms, *SDQ* Strength and Difficulties Questionnaire, *CU* Callous-Unemotional.

**Table 2:** Sample characteristics for the CP improvers, Persisters and Control group (Improvers and Non-improvers groups based on PACS CP scores).

		Mean (SD)		*F*	*p*
	Controls	CP Persisters	CP Improvers		
*n*	43	31	32		
Age (months)	101.80 (18.35)	104.0 (20.41)	102.93 (16.01)	0.124	*p* = 0.88
IQ	109.16 (15.5)	100.43 (14.05)	105.19 (13.47	3.2	*p* = 0.045
Handedness	−6.72 (3.68)	−5.44 (6.95)	−7.0 (4.40)	0.647	*p* = 0.52
SES (Maternal Education)	5.68 (2.33)	4.51 (2.59)	3.89 (2.67)	4.52	*p* = 0.01
ADHD	16.10 (9.79)	52.24 (16.92)	55.53 (13.78)	101.51	*p* < 0.001
Motion in Scanner	0.36 (0.29)	0.41 (0.32)	0.47 (0.36)	1.13	*p* = 0.32
Days between T1 & T2 scans	122.62 (27.63)	123.29 (36.4)	127.43 (36.75)	0.21	*p* = 0.81

*CP* Conduct Problems, *IQ* Intelligence Quotient, *SES* socio-economic status, *ADHD* attention deficit hyperactivity disorder, *T1* Timepoint 1, *T2* Timepoint 2, *SD* standard deviation.

### Clinical data

The children with CP had significantly reduced CP symptoms (PACS) following intervention (Pre: 1.55 ± 0.55, Post: 1.36 ± 0.56; *F*_(1,63.19)_ = 13.03, *p* < 0.001, η2 = 0.17). There was also a significant reduction in ADHD symptoms (Pre: 54.00 ± 2.18, Post: 50.13 ± 2.17; *F*_(1,63.09)_ = 5.44, *p* = 0.023, η2 = 0.08) and CU traits scores (Pre: 34.35 ± 1.39, Post: 30.77 ± 1.39; *F*_(1,186.89)_ = 34.36, *p* < 0.001, η2 = 0.16) observed in the CP group following the intervention.

We then examined any differences in behaviour and symptom change between the improving and persistent CP groups. A significant *group-by-time* interaction was observed for CP symptoms (*F*_1,61)_ = 91.662, *p* < 0.001, η2 = 0.6), but not ADHD (*F*_1,58.22)_ = 1.981, *p* = 0.165, η2 = 0.03) or CU traits scores (*F*_1,58.616)_ = 2.158, *p* = 0.147, η2 = 0.04) (Table 3).

**Table 3:** Behavioural Scores for the Improving and persistent CP groups.

	Mean (SE)		*F*	*P*^*a*^
	CP Persisters	CP Improvers		
CP (PACS) T1	1.38 (0.75)	1.76 (0.74)	91.66	*p* < 0.001
CP (PACS) T2	1.51 (0.75)	1.23 (0.74)		
CP (SDQ) T1	5.33 (0.42)	5.65 (0.41)	5.54	*p* = 0.022
CP (SDQ) T2	4.82 (0.42)	4.03 (0.40)		
CU traits T1	32.26 (2.21)	36.21 (2.15)	2.15	*p* = 0.147
CU traits T2	30.25 (2.22)	31.10 (2.14)		
ADHD T1	52.06 (3.12)	55.80 (3.02)	1.98	*p* = 0.165
ADHD T2	50.87 (3.12)	49.72 (2.99)		

*CP* Conduct Problems, *PACS* Parental Account of Children’s Symptoms, *SDQ* Strength and Difficulties Questionnaire, *CU* Callous-Unemotional.

^a^p-value represents the group-by-time results.

### DTI tractography

**Fig. 2:**
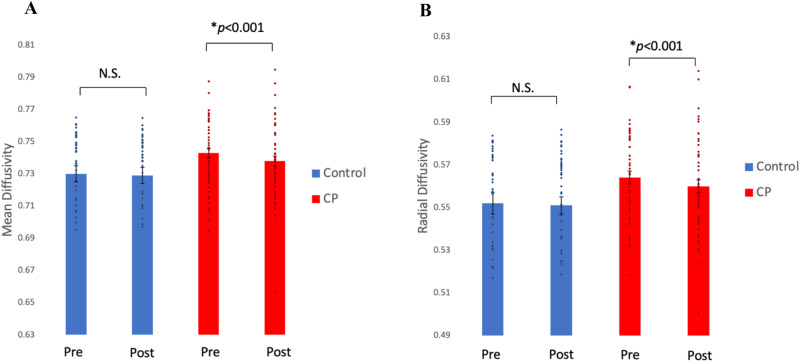
Changes in white matter microstructure between two groups over time. Measures of (**A**) mean diffusivity and (**B**) radial diffusivity in the uncinate fasciculus pre and post a parenting intervention in the Conduct Problem (CP) group compared to the control group.

We found a statistically significant change in white matter microstructure of the UF in the CP group following the parenting programme. A significant *group-by-time* interaction was found in both hemispheres between the CP group and TD controls with respect to MD (*F*_(1,282.38)_ = 5.04, *p* = 0.026) and RD (*F*_(1,282.33)_ = 6.36, *p* = 0.012). FDR adjustment was applied to RD, the independent measure, which survived multiple correction (*q* = 0.03). Post hoc tests revealed that prior to the intervention the CP group had significantly increased MD (*p* = 0.034) and RD (*p* = 0.044) in the UF compared to the control group. However, after the intervention there was a significant decrease in MD (*p* < 0.001) and RD (*p* < 0.001), across hemispheres (i.e., following intervention the CP group more closely resembled the control group (Fig. 2)). There were no significant microstructural changes observed in the control group over time (Fig. 2). Post hoc tests also revealed following the intervention, there were no longer significant differences observed in RD (*p* = 0.308) or MD (*p* = 0.228) between the CP and control groups.

There were no significant *group-by-time* interactions observed for FA (*F*_(1,277.81)_ = 3.72, *p* = 0.055) or AD (*F*_(1,282.83)_ = 2.01, *p* = 0.157) when comparing the CP group and TD control group. Further, between the CP and TD control groups, there were no main effects of group observed for MD (*F*_(1,93.79)_ = 2.60, *p* = 0.110), RD (*F*_(1,93.89)_ = 2.17, *p* = 0.144), FA (*F*_(1,95.43)_ = 1.03, *p* = 0.312) or AD (*F*_(1,93.52)_ = 2.88, *p* = 0.093) overall across timepoints. Finally, between the CP group and TD control group a significant *hemisphere-by-group* interaction was found for FA (*F*_(1,276.15)_ = 3.72, *p* = 0.034)—see supplementary material (Fig S1).

However, when testing our second hypothesis that reduction in UF abnormalities would be greatest in ‘CP improvers’, compared to ‘CP persisters’ or TD controls, there was no significant *group-by-time* effect for any DTI measure.

### Model comparison

In the absence of an association between changes in UF microstructure and CP symptom reduction (Hypothesis 2), we explored the relationship between changes in UF microstructure and changes in ADHD and CU traits (i.e., as they also decreased in the CP group post treatment). Therefore, using the same MICD approach as before, *‘improvers’* and ‘*persisters’* were respectively defined by the presence or absence of a 0.4 SD reduction in ADHD or CU traits.

Using a likelihood-ratio test, the predictive value of these ‘response’ variables (i.e., ADHD and CU) were then compared to determine which best explained the observed changes in UF microstructure data. The predictive value of the model only increased, when change in CU traits was used as the grouping variable—see supplementary material (Table S1*)*. Therefore, we completed *post hoc* analyses to explore whether brain changes associated with CU symptoms might explain the overall pattern we observed in the CP group (i.e., the significant changes observed in MD and RD following the intervention). For completeness the sample characteristics for ADHD improvers/persisters and CU improvers/persisters have been included in the supplementary material—(see Table S2 and Table S3*)*.

### Post hoc analysis

**Fig. 3:**
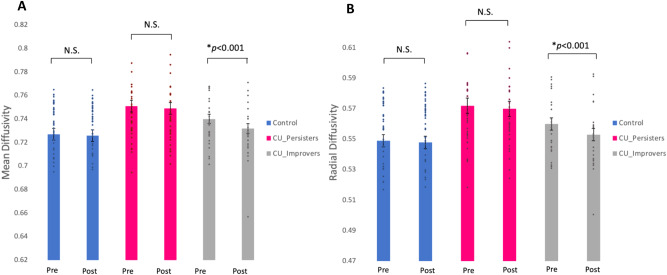
Changes in white matter microstructure between three groups over time. Measures of microstructural integrity pre and post a parenting intervention in a typically developing control group compared to CU traits improvers and persisters in the uncinate fasciculus for (**A**) mean diffusivity and (**B**) radial diffusivity.

In an exploratory *post hoc* analysis (i.e. UF abnormalities will remain fixed in children with CP with persistent CU traits versus improved CU traits) we observed a significant *group-by-time* interaction for MD (*F*_(2,276.54)_ = 6.90, *p* = 0.001) and RD (*F*_(2,276.49)_ = 6.65, *p* = 0.002) (which survived FDR adjustment *q* = 0.02). *Post hoc* tests suggested that the decrease in diffusion measures in the CP group post intervention was driven by those whose CU traits improved (Fig. 3). There were no significant microstructural changes in the UF observed over time in the CU persisters group or the TD control group.

There was also a significant main effect of group observed for MD (*F*_(2,91.58)_ = 5.32, *p* = 0.006) and RD (*F*_(2,91.69)_ = 5.41, *p* = 0.006) (which survived FDR adjustment *q* = 0.03) with the CU persisters showing significantly increased MD and RD overall across hemisphere and across both timepoints (Fig S2—supplementary material).

Finally in the CP group, 52/67 boys were not prescribed any medication, 7/67 boys, were prescribed ADHD medication, and 8/67 children had an unknown medication history. We subsequently re-ran all our analyses, initially excluding the 7/67 boys on medication, and then also excluding the 8/67 boys with unknown medication history. We found that all our results remained significant.

## Discussion

Consistent with prior studies, we found significant anatomical differences in the UF of children with CP, compared with TD controls. However, for the first time, we also report that these differences are not fixed, but ‘normalise’ following a parenting intervention. For example, following the intervention, UF diffusivity (MD and RD) significantly reduced in children with CP (i.e., in the direction of TD controls). Further, over the same period, UF microstructure remained fixed in TD children, suggesting that the changes observed in the CP group were due to the intervention rather than maturational effects.

*Post hoc* analyses in the CP group were also used to better understand the relationship between changes in UF microstructure and behavioural change. Contrary to our a priori hypothesis, this relationship was not directly driven by change in PACS score (i.e., a measure of the frequency and severity of antisocial behaviours) but by change in ICU score (i.e., a measure of callousness, uncaring, and unemotional behaviour). Prior studies have reported reduced CU traits following targeted parenting intervention [60] and changes in parenting style [61, 62], particularly in younger children [63–65]. However, we had hypothesized that UF change would be observed in those whose CP improved, rather than being associated with improvement in CU traits, as the latter tends to be more resistant to treatment. A possible explanation for this finding is that behavioural changes measured by the PACS are associated with microstructural changes in other tract(s). Whilst previous studies supported the a priori approach to focusing on the UF, we recognise that this tract forms part of a larger brain network, and this will need to be explored in future studies.

Our findings are however in line with previous DTI studies which have reported that UF microstructural abnormalities are associated with CU traits rather than CP more generally (though these findings have not always been replicated) [25, 26, 28, 36, 66–68]. We provide longitudinal data to support this relationship. Specifically, we show that individuals whose CU traits improve over time, also show concomitant changes in UF microstructure. Interestingly, altered diffusion parameters were also observed prior to the intervention in those whose CU persisted. This may therefore play a role in predicting outcome in future studies. However, it is likely that successful outcome prediction using supervised learning will require multiple sources of information (e.g., clinical, and multimodal neuroimaging / computational data).

Our results are also consistent with other DTI studies reporting rapid changes in white matter microstructure in response to short-term training. For instance, large-scale changes in MD in several white matter tracts were observed in children who completed an 8-week intensive reading intervention, demonstrating that controlled changes to a child’s educational environment can induce significant changes in white matter [69]. In addition, an increase in FA was reported in healthy adults following a 6-week training intervention of learning a complex visuo-motor skill [70]. These microstructural changes in healthy adults were still detectable four weeks later without any intervention in-between suggesting that white matter changes may be long lasting.

While the current study was not designed to explore what is happening at the microscopic level, the observed rapid changes in white matter microstructure may arise from several mechanisms including changes in myelination [71]. White matter consists of bundles of axonal fibres which are surrounded by myelin sheaths to a varying degree. Myelin, which is produced by glial cells known as oligodendrocytes, has previously been thought of as a more permanent feature in the brain, responsible for axon conductance, however there is now considerable evidence suggesting that myelination plays an important role in plasticity [72]. For example, an increase in neuronal activity (such as an increase in concentrated learning) is linked with the generation of new oligodendrocytes, which subsequently enhances myelin formation in the brain [73, 74]. Therefore, in the current study, it is plausible that boys with CP have enhanced neuronal activity following the parenting intervention, which results from learning and implementing new positive forms of behaviour. Firstly, this may stimulate the rapid growth of new myelinating oligodendrocytes in the UF, which is thought to be reflected by a reduction in MD [69], as reported in the current study. Secondly, it is likely that this increase in oligodendrocytes is followed by increased myelination, which is associated with a rapid decrease in RD [75]—also reported in the current study. Therefore, our findings indicate that learning to adapt to new parenting strategies may lead to regionally specific plasticity in white matter tracts, such as the UF.

Future studies are also needed to understand why a subgroup of children with CP with high CU traits did not improve following intervention. This could be due to several reasons that relate to both boys with CP and their parents. For example, previous studies have reported treatment outcome may be influenced by parental engagement with the intervention and relationship with the program facilitator [76, 77]. Some parents may also require a ‘personalised’ program (e.g., a one-to-one, and/or home-based approach) that is more tailored to their specific needs [78, 79]. This is currently being explored in a subgroup of boys from our study who failed to respond to the group parenting program. Also, CU traits can be fractionated into distinct subtypes, which may have variable susceptibility to change in response to group or personalised interventions. Primary CU traits, for example, refers to those with a greater genetic underpinning, are associated with abnormalities in the oxytocin system [80], and underpinned by deficits in emotion processing [81]. However, secondary CU traits refer to those that are more related to environmental factors, such as parental rejection and childhood trauma [81]. Therefore, the latter variant may be more susceptible to modification following the parenting intervention, whereas the primary subtype may be better targeted with a pharmacological approach (e.g., modulation of the oxytocinergic system).

Although the current study has many strengths, it is not without limitations. The primary aim of the study was to explore whether abnormal UF microstructure in children with CP was fixed or reversible if CP symptoms reduced. Whilst our study was well-designed to address this question, this study was not a clinical trial and the absence of a subgroup of children with CP who were unexposed to parenting intervention, limited the strength of secondary inferences regarding the link between treatment and changes in CU traits. However, CU traits are very stable, and it is extremely unlikely that the changes observed in our clinical sample would have occurred naturally over such a short period [82]. In addition to including a further control group, future studies might also benefit from expanding the range of tracts analysed. UF abnormalities associated with ASB are often considered to be more robust than aberrations in other tracts. However, our group [60, 83] and others (see [36] for review) have increasingly reported consistent changes in a wider network of tracts in this population, and these should be included in future exploration. A further limitation of the current study is that the sample consisted of male participants only. In recent years several studies have identified differences in brain structure and function between male and female youth with CP [21, 84, 85] therefore, future longitudinal studies which include both genders are warranted, to investigate if female children with CP also show microstructural changes in the UF following a significant change in behaviour. Finally, it should be acknowledged that while previous studies have provided evidence that the sample size in the current study is large enough to detect group differences in white matter microstructure in this cohort [36], further studies with larger samples sizes are recommended to replicate our results.

Although the effects of group parenting programs have been widely studied [33], there has been lack of research investigating if parenting programs have a significant effect on brain structure. Our study provided evidence for the first time that parenting interventions may have an effect, not only at the behavioural level but also at the neural level. These results may have important implications for policymaking and commissioning in providing resources to ensure such interventions remain funded and continue to be accessible to children who are most at-risk for developing ASB. In summary, our study shows that the CP group had significant microstructural changes in UF following a ‘gold-standard’ parenting program, such that their white matter microstructure more closely resembled that of TD control boys. Interestingly, these microstructural changes were associated with reduction in CU traits and *not* with a reduction in CP symptoms. Our findings suggest a link between CU traits and abnormalities in the UF development, as well as the plasticity of white matter tracts in response to early intervention. Advancing our understanding of the neural mechanisms underlying response or persistence of ASB following parenting intervention, could provide putative biomarkers for the development of future treatment options.

### Supplementary information

Supplementary material

Fig S1

Fig S2

## References

[1] Office for National Statistics. Mental health of children and young people in Great Britain. 2004; Available from: https://files.digital.nhs.uk/publicationimport/pub06xxx/pub06116/ment-heal-chil-youn-peop-gb-2004-rep1.pdf.

[2] Piquero AR, Shepherd I, Shepherd JP, Farrington DP (2011). Impact of offending trajectories on health: Disability, hospitalisation and death in middle-aged men in the Cambridge Study in Delinquent Development. Crim Behav Ment Health.

[3] Odgers CL, Caspi A, Broadbent JM, Dickson N, Hancox RJ, Harrington HL (2007). Prediction of differential adult health burden by conduct problem subtypes in males. Arch Gen Psychiatry.

[4] Rivenbark JG, Odgers CL, Caspi A, Harrington HL, Hogan S, Houts RM (2018). The high societal costs of childhood conduct problems: evidence from administrative records up to age 38 in a longitudinal birth cohort. J Child Psychol Psychiatry.

[5] Moffitt TE, Caspi A, Harrington H, Milne BJ (2002). Males on the life-course-persistent and adolescence-limited antisocial pathways: follow-up at age 26 years. Dev Psychopathol.

[6] Frick PJ, Viding E (2009). Antisocial behavior from a developmental psychopathology perspective. Dev Psychopathol.

[7] Scott S, O’Connor TG, Futh A, Matias C, Price J, Doolan M (2010). Impact of a parenting program in a high-risk, multi-ethnic community: The PALS trial. J Child Psychol Psychiatry.

[8] Scott S, Sylva K, Doolan M, Price J, Jacobs B, Crook C (2010). Randomised controlled trial of parent groups for child antisocial behaviour targeting multiple risk factors: the SPOKES project. J Child Psychol Psychiatry.

[9] Scott S, Spender Q, Doolan M, Jacobs B, Aspland H (2001). Multicentre controlled trial of parenting groups for childhood antisocial behaviour in clinical practice. Br Med J.

[10] Scott S, Sylva K, Beckett C, Kallitsoglou A, Doolan M, Ford T (2012). Should parenting programmes to improve children’s life chances address child behaviour, reading skills, or both? Rationale for the helping children achieve trial.. Eur J Dev. Psychol.

[11] Scott S, Briskman J, O’Connor TG (2014). Early prevention of antisocial personality: long-term follow-up of two randomized controlled trials comparing indicated and selective approaches. Am J Psychiatry.

[12] Scott S (2005). Do parenting programmes for severe child antisocial behaviour work over the longer term, and for whom? One year follow-up of a multi-centre controlled trial. Behav Cogn Psychother.

[13] Dandreaux DM, Frick PJ (2009). Developmental Pathways to conduct problems: a further test of the childhood and adolescent-onset distinction. J Abnorm Child Psychol.

[14] Frick PJ, Ray JV, Thornton LC, Kahn RE (2014). Can callous-unemotional traits enhance the understanding, diagnosis, and treatment of serious conduct problems in children and adolescents? A comprehensive review. Psychol Bull.

[15] Catani M, Howard RJ, Pajevic S, Jones DK (2002). Virtual in Vivo interactive dissection of white matter fasciculi in the human brain. Neuroimage.

[16] Kier EL, Staib LH, Davis LM, Bronen RA (2004). MR imaging of the temporal stem: anatomic dissection tractography of the uncinate fasciculus, inferior occipitofrontal fasciculus, and Meyer’s loop of the optic radiation. Am J Neuroradiol.

[17] Bhatia K, Henderson L, Yim M, Hsu E, Dhaliwal R (2018). Diffusion tensor imaging investigation of uncinate fasciculus anatomy in healthy controls: description of a subgenual stem. Neuropsychobiology.

[18] Blair RJR (2008). Review. The amygdala and ventromedial prefrontal cortex: functional contributions and dysfunction in psychopathy. Philos Trans R Soc B: Biol Sci.

[19] de Oliveira-Souza R, Hare RD, Bramati IE, Garrido GJ, Azevedo Ignácio F, Tovar-Moll F (2008). Psychopathy as a disorder of the moral brain: fronto-temporo-limbic grey matter reductions demonstrated by voxel-based morphometry. Neuroimage.

[20] Raine A, Yang Y (2006). Neural foundations to moral reasoning and antisocial behavior. Soc Cogn Affect Neurosci.

[21] Rogers JC, Gonzalez-Madruga K, Kohls G, Baker RH, Clanton RL, Pauli R (2019). White matter microstructure in youths with conduct disorder: effects of sex and variation in callous traits. J Am Acad Child Adolesc Psychiatry.

[22] Marsh AA, Finger EC, Fowler KA, Adalio CJ, Jurkowitz ITN, Schechter JC (2013). Empathic responsiveness in amygdala and anterior cingulate cortex in youths with psychopathic traits. J Child Psychol Psychiatry.

[23] Raine A (2011). An amygdala structural abnormality common to two subtypes of conduct disorder: a neurodevelopmental conundrum. Am J Psychiatry.

[24] Waller R, Gard AM, Shaw DS, Forbes EE, Neumann CS, Hyde LW (2019). Weakened functional connectivity between the amygdala and the ventromedial prefrontal cortex is longitudinally related to psychopathic traits in low-income males during early adulthood. Clin Psychol Sci.

[25] Graziano PA, Garic D, Dick AS (2022). Individual differences in white matter of the uncinate fasciculus and inferior fronto-occipital fasciculus: possible early biomarkers for callous-unemotional behaviors in young children with disruptive behavior problems. J Child Psychol Psychiatry.

[26] Sarkar S, Craig MC, Catani M, Dell’Acqua F, Fahy T, Deeley Q (2013). Frontotemporal white-matter microstructural abnormalities in adolescents with conduct disorder: a diffusion tensor imaging study. Psychol Med.

[27] Passamonti L, Fairchild G, Fornito A, Goodyer IM, Nimmo-Smith I, Hagan CC (2012). Abnormal anatomical connectivity between the amygdala and orbitofrontal cortex in conduct disorder. PLoS ONE.

[28] Zhang J, Gao J, Shi H, Huang B, Wang X, Situ W (2014). Sex differences of uncinate fasciculus structural connectivity in individuals with conduct disorder. Biomed Res Int.

[29] Craig MC, Catani M, Deeley Q, Latham R, Daly E, Kanaan R (2009). Altered connections on the road to psychopathy. Mol Psychiatry.

[30] Sobhani M, Baker L, Martins B, Tuvblad C, Aziz-Zadeh L (2015). Psychopathic traits modulate microstructural integrity of right uncinate fasciculus in a community population. Neuroimage Clin.

[31] Wolf RC, Pujara MS, Motzkin JC, Newman JP, Kiehl KA, Decety J (2015). Interpersonal traits of psychopathy linked to reduced integrity of the uncinate fasciculus. Hum Brain Mapp.

[32] Motzkin JC, Newman JP, Kiehl KA, Koenigs M (2011). Reduced prefrontal connectivity in psychopathy. J Neurosci.

[33] Leijten P, Gardner F, Landau S, Harris V, Mann J, Hutchings J (2018). Research Review: Harnessing the power of individual participant data in a meta-analysis of the benefits and harms of the Incredible Years parenting program. J Child Psychol Psychiatry.

[34] Sanders MR, Kirby JN, Tellegen CL, Day JJ (2014). The Triple P-Positive Parenting Program: a systematic review and meta-analysis of a multi-level system of parenting support. Clin Psychol Rev.

[35] Goodman R (1997). The strengths and difficulties questionnaire: a research note. J Child Psychol Psychiatry.

[36] Waller R, Dotterer HL, Murray L, Maxwell AM, Hyde LW (2017). White-matter tract abnormalities and antisocial behavior: a systematic review of diffusion tensor imaging studies across development. Neuroimage Clin.

[37] Taylor E, Chadwick O, Heptinstall E, Danckaerts M (1996). Hyperactivity and conduct problems as risk factors for adolescent development. J Am Acad Child Adolesc Psychiatry.

[38] Norman GR, Sloan JA, Wyrwich KW (2003). Interpretation of changes in health-related quality of life. Med Care.

[39] Jaeschke R, Singer J, Guyatt GH (1989). Measurement of health status. ascertaining the minimal clinically important difference. Control Clin Trials.

[40] Collaborating Centre for Mental Health. The NICE guidelines on antisocial behaviour and conduct disorders in children and young people: recognition and management. British Psychological Society. 2013. Available from: http://www.nice.org.uk/guidance/cg158. 26065062

[41] Kimonis ER, Frick PJ, Skeem JL, Marsee MA, Cruise K, Munoz LC (2008). Assessing callous-unemotional traits in adolescent offenders: validation of the Inventory of Callous-Unemotional Traits. Int J Law Psychiatry.

[42] Conners CK. Conners 3rd Edition (Conners 3). Journal of Psychoeducational Assessment. 2008.

[43] Wechsler D. Manual for the Wechsler abbreviated intelligence scale (WASI). San Antonio, TX: Psychological Corporation. 1999.

[44] Annett M (1970). A classification of hand preference by association analysis. Br J Psychol.

[45] Veraart J, Fieremans E, Novikov DS (2016). Diffusion MRI noise mapping using random matrix theory. Magn Reson Med.

[46] Kellner E, Dhital B, Kiselev VG, Reisert M (2016). Gibbs-ringing artifact removal based on local subvoxel-shifts. Magn Reson Med.

[47] Pierpaoli C, Walker L, Irfanoglu M, Barnett A, Basser P, Chang LC, et al. TORTOISE: an integrated software package for processing of diffusion MRI data. ISMRM 18th annual meeting 2010, Stockholm, Sweden. 2010.

[48] Irfanoglu MO, Nayak A, Jenkins J, Pierpaoli C. TORTOISE v3: Improvements and new features of the NIH diffusion MRI processing pipeline. In: The ISMRM 25th Annual Meeting & Exhibition. 2017.

[49] Andersson JLR, Skare S, Ashburner J (2003). How to correct susceptibility distortions in spin-echo echo-planar images: application to diffusion tensor imaging. Neuroimage.

[50] Andersson JLR, Sotiropoulos SN (2016). An integrated approach to correction for off-resonance effects and subject movement in diffusion MR imaging. Neuroimage.

[51] Andersson JLR, Graham MS, Zsoldos E, Sotiropoulos SN (2016). Incorporating outlier detection and replacement into a non-parametric framework for movement and distortion correction of diffusion MR images. Neuroimage.

[52] Andersson JLR, Graham MS, Drobnjak I, Zhang H, Filippini N, Bastiani M (2017). Towards a comprehensive framework for movement and distortion correction of diffusion MR images: within volume movement. Neuroimage.

[53] Basser PJ, Pajevic S, Pierpaoli C, Duda J, Aldroubi A (2000). In vivo fiber tractography using DT-MRI data. Magn Reson Med.

[54] Dell’Acqua F, Lacerda L, Catani M, Simmons A. Anisotropic Power Maps: A diffusion contrast to reveal low anisotropy tissues from HARDI data. In Proceedings Joint Annual Meeting ISMRM/ESMRMB, (Milan). 2013.

[55] Dell’Acqua F, Lacerda L, Barrett R, D’Anna L, Tsermentseli S, Goldstein L (2015). Megatrack: a fast and effective strategy for group comparison and supervised analysis of large-scale tractography datasets. Proc Int Soc Magn Reson Med.

[56] Avants BB, Tustison N, Johnson H. Advanced Normalization Tools (ANTS) Release 2.x. 2014. Available from: https://brianavants.wordpress.com/2012/04/13/updated-ants-compile-instructions-april-12-2012/.

[57] Catani M, Thiebaut de Schotten M (2008). A diffusion tensor imaging tractography atlas for virtual in vivo dissections. Cortex.

[58] Benjamini Y, Hochberg Y (1995). Controlling the false discovery rate: a practical and powerful approach to multiple testing. Source: J R Stat Soc Ser B (Methodol).

[59] Scholz J, Tomassini V, Johansen-Berg H. Individual Differences in White Matter Microstructure in the Healthy Brain. In: Diffusion MRI: From Quantitative Measurement to In vivo Neuroanatomy: Second Edition. Elsevier Inc. 2013. p. 301–16.

[60] Sethi A, Sarkar S, Dell’Acqua F, Viding E, Catani M, Murphy DGM (2018). Anatomy of the dorsal default-mode network in conduct disorder: association with callous-unemotional traits. Dev Cogn Neurosci.

[61] Muratori P, Milone A, Manfredi A, Polidori L, Ruglioni L, Lambruschi F (2017). Evaluation of improvement in externalizing behaviors and callous-unemotional traits in children with disruptive behavior disorder: a 1-year follow up clinic-based study. Adm Policy Ment Health Ment Health Serv Res.

[62] Hawes DJ, Dadds MR, Frost ADJ, Hasking PA (2011). Do childhood callous-unemotional traits drive change in parenting practices?. J Clin Child Adolesc Psychol.

[63] Fanti KA, Munoz Centifanti LC (2014). Childhood callous-unemotional traits moderate the relation between parenting distress and conduct problems over time. Child Psychiatry Hum Dev.

[64] Hawes DJ, Dadds MR (2007). Stability and malleability of callous-unemotional traits during treatment for childhood conduct problems. J Clin Child Adolesc Psychol.

[65] Somech LY, Elizur Y (2012). Promoting self-regulation and cooperation in pre-kindergarten children with conduct problems: a randomized controlled trial. J Am Acad Child Adolesc Psychiatry.

[66] McDonald R, Dodson MC, Rosenfield D, Jouriles EN (2011). Effects of a parenting intervention on features of psychopathy in children. J Abnorm Child Psychol.

[67] Villemonteix T, Rogers JC, Courbet O, Gonzalez-Madruga K, Kohls G, Raschle NM (2021). Sex matters: association between callous-unemotional traits and uncinate fasciculus microstructure in youths with conduct disorder. Brain Imaging Behav.

[68] Puzzo I, Seunarine K, Sully K, Darekar A, Clark C, Sonuga-Barke EJS (2018). Altered white-matter microstructure in conduct disorder is specifically associated with elevated callous-unemotional traits. J Abnorm Child Psychol.

[69] Breeden AL, Cardinale EM, Lozier LM, VanMeter JW, Marsh AA (2015). Callous-unemotional traits drive reduced white-matter integrity in youths with conduct problems. Psychol Med.

[70] Huber E, Donnelly PM, Rokem A, Yeatman JD (2018). Rapid and widespread white matter plasticity during an intensive reading intervention. Nat Commun.

[71] Scholz J, Klein MC, Behrens TEJ, Johansen-Berg H (2009). Training induces changes in white matter architecture. Nat Neurosci.

[72] Walhovd KB, Johansen-Berg H, Káradóttir RT (2014). Unraveling the secrets of white matter – Bridging the gap between cellular, animal and human imaging studies. Neuroscience.

[73] Xin W, Chan JR (2020). Myelin plasticity: sculpting circuits in learning and memory. Nat Rev Neurosci.

[74] Gibson EM, Purger D, Mount CW, Goldstein AK, Lin GL, Wood LS (2014). Neuronal activity promotes oligodendrogenesis and adaptive myelination in the mammalian brain. Science.

[75] Hughes EG, Orthmann-Murphy JL, Langseth AJ, Bergles DE (2018). Myelin remodeling through experience-dependent oligodendrogenesis in the adult somatosensory cortex. Nat Neurosci.

[76] Winklewski PJ, Sabisz A, Naumczyk P, Jodzio K, Szurowska E, Szarmach A (2018). Understanding the physiopathology behind axial and radial diffusivity changes-what do we Know?. Front Neurol.

[77] Koerting J, Smith E, Knowles MM, Latter S, Elsey H, McCann DC (2013). Barriers to, and facilitators of, parenting programmes for childhood behaviour problems: a qualitative synthesis of studies of parents’ and professionals’ perceptions. Eur Child Adolesc Psychiatry.

[78] Kazdin AE, Whitley MK (2006). Pretreatment social relations, therapeutic alliance, and improvements in parenting practices in parent management training. J Consult Clin Psychol.

[79] Niec LN, Barnett ML, Prewett MS, Shanley Chatham JR (2016). Group Parent-Child Interaction Therapy: A Randomized Control Trial for the Treatment of Conduct Problems in Young Children. J Consult Clin Psychol.

[80] McKay K, Kennedy E, Senior R, Scott S, Hill J, Doolan M (2020). Informing the personalisation of interventions for parents of children with conduct problems: a qualitative study. BMC Psychiatry.

[81] Fragkaki I, Verhagen M, van Herwaarden AE, Cima M (2019). Daily oxytocin patterns in relation to psychopathy and childhood trauma in residential youth. Psychoneuroendocrinology.

[82] Craig SG, Goulter N, Moretti MM (2020). A systematic review of primary and secondary callous-unemotional traits and psychopathy variants in youth. Clin Child Fam Psychol Rev.

[83] Frick PJ, Stickle TR, Dandreaux DM, Farrell JM, Kimonis ER (2005). Callous-unemotional traits in predicting the severity and stability of conduct problems and delinquency. J Abnorm Child Psychol.

[84] Sethi A, Gregory S, Dell’Acqua F, Periche Thomas E, Simmons A, Murphy DGM (2015). Emotional detachment in psychopathy: involvement of dorsal default-mode connections. Cortex.

[85] Fairchild G, Hagan CC, Walsh ND, Passamonti L, Calder AJ, Goodyer IM (2013). Brain structure abnormalities in adolescent girls with conduct disorder. J Child Psychol Psychiatry.

