# Supplementary material for: Rapid white matter changes in children with conduct problems during a parenting intervention
Source: Transl Psychiatry. 2023 Nov 4;13:339. doi: 10.1038/s41398-023-02635-8 (PMC10625622; doi:10.1038/s41398-023-02635-8)
Supplement: Supplementary file 1 — Supplementary material [file 41398_2023_2635_MOESM1_ESM.docx]

**Supplementary material**

*Fig S1. Hemisphere*Group interaction for fractional anisotropy (FA) between the conduct problem (CP) and control group in the uncinate fasciculus*

*Fig S2. Effect of ‘change in callous-unemotional (CU) traits’ on white matter microstructure (Exploratory post hoc analysis). Graphs display the main effects of group in the uncinate fasciculus for A) mean diffusivity (RD) and B) radial diffusivity (MD) between the CU improvers, CU persisters and controls. FDR adjustment was applied to RD which survived multiple comparisons (q=0.03)*

*Table S1: Goodness of Fit – values represent improvers/persisters groupings (for conduct problems (CP), attention deficit hyperactivity disorder (ADHD), and callous-unemotional (CU) traits) when compared to the overall CP versus Controls groupings.*

|  | **AIC** | **BIC** | ***Chisq*** | **Df** | ***p*** |
| --- | --- | --- | --- | --- | --- |
| **UF – Mean Diffusivity** |  |  |  |  |  |
| CP response | -2255.9 | -2181.6 | 7.76 | 4 | 0.100 |
| ADHD response | -2255.0 | -2180.7 | 6.89 | 4 | 0.141 |
| CU response | -2266.9 | -2192.9 | 18.83 | 4 | *p*<0.001*** |
| **UF – Radial Diffusivity** |  |  |  |  |  |
| CP response | -2292.3 | -2218.0 | 6.78 | 4 | *p*=0.147 |
| ADHD response | -2293.9 | -2219.7 | 8.39 | 4 | *p*=0.078 |
| CU response | -2302.9 | -2228.7 | 17.43 | 4 | *p=*0.001** |

*Table S2: Sample Characteristics for the CU improvers, Persisters and Control group (Improvers and Non-improvers groups based on ICU scores)*

|  |  | **Mean (SD)** |  | ***F*** | ***p*** |
| --- | --- | --- | --- | --- | --- |
|  | **Controls** | **Persisters** | **Improvers** |  |  |
| ***n*** | 43 | 31 | 32 |  |  |
| **Age (months)** | 101.80 (18.35) | 102.03 (19.08) | 103.96 (16.02) | 0.131 | *p*=0.87 |
| **IQ** | 109.16 (15.5) | 101.55 (14.10) | 104.62 (13.49) | 2.66 | *p*=0.07 |
| **Handedness** | -6.72 (3.68) | -3.65 (7.0) | -8.03 (4.28) | 4.83 | *p*=0.01 |
| **SES (Maternal Education)** | 5.68 (2.33) | 4.06 (2.47) | 4.21 (2.87) | 4.46 | *p*=0.01 |
| **ADHD** | 16.10 (9.79) | 59.77 (12.27) | 47.72 (17.94) | 112.18 | *p*<0.001 |
| **Motion in Scanner** | 0.36 (0.29) | 0.49 (0.34) | 0.38 (0.32) | 1.69 | *p*=0.18 |
| **Days between T1 & T2 scans** | 122.62 (27.63) | 117.65 (38.68) | 128.34 (32.70) | 0.83 | *p*=0.43 |

CP = Conduct Problems; IQ = Intelligence Quotient; SES = Socio-Economic Status; ADHD = attention deficit hyperactivity disorder; T1 = Timepoint 1; T2 = Timepoint 2; SD = Standard Deviation

*Table S3: Sample Characteristics for the ADHD improvers, Persisters and Control group (Improvers and Non-improvers groups based on Conners ADHD scores)*

|  |  | **Mean (SD)** |  | ***F*** | ***p*** |
| --- | --- | --- | --- | --- | --- |
|  | **Controls** | **Persisters** | **Improvers** |  |  |
| ***n*** | 43 | 41 | 26 |  |  |
| **Age (months)** | 101.80 (18.35) | 101.71 (18.46) | 105.28 (17.67) | 0.35 | *p*=0.702 |
| **IQ** | 109.16 (15.5) | 103.12 (11.20) | 102.08 (17.26) | 2.58 | *p*=0.080 |
| **Handedness** | -6.72 (3.68) | -5.82 (6.28) | -6.10 (5.82) | 0.19 | *p*=0.823 |
| **SES (Maternal Education)** | 5.68 (2.33) | 4.27 (2.62) | 4.04 (2.75) | 4.25 | *p*=0.017 |
| **ADHD at T1** | 16.10 (9.79) | 51.09 (17.0) | 58.39 (14.24) | 98.48 | *p*<0.001 |
| **Motion in Scanner** | 0.36 (0.29) | 0.41 (0.30) | 0.50 (0.38) | 1.73 | *p*=0.182 |
| **Days between T1 & T2 scans** | 122.62 (27.63) | 127.14 (34.40) | 119.84 (41.12) | 0.405 | *p*=0.668 |
