# Supplementary material for: Rapid white matter changes in children with conduct problems during a parenting intervention
Source: Transl Psychiatry. 2023 Nov 4;13:339. doi: 10.1038/s41398-023-02635-8 (PMC10625622; doi:10.1038/s41398-023-02635-8)
Supplement: Supplementary file 2 — Fig S1 [file 41398_2023_2635_MOESM2_ESM.pdf]

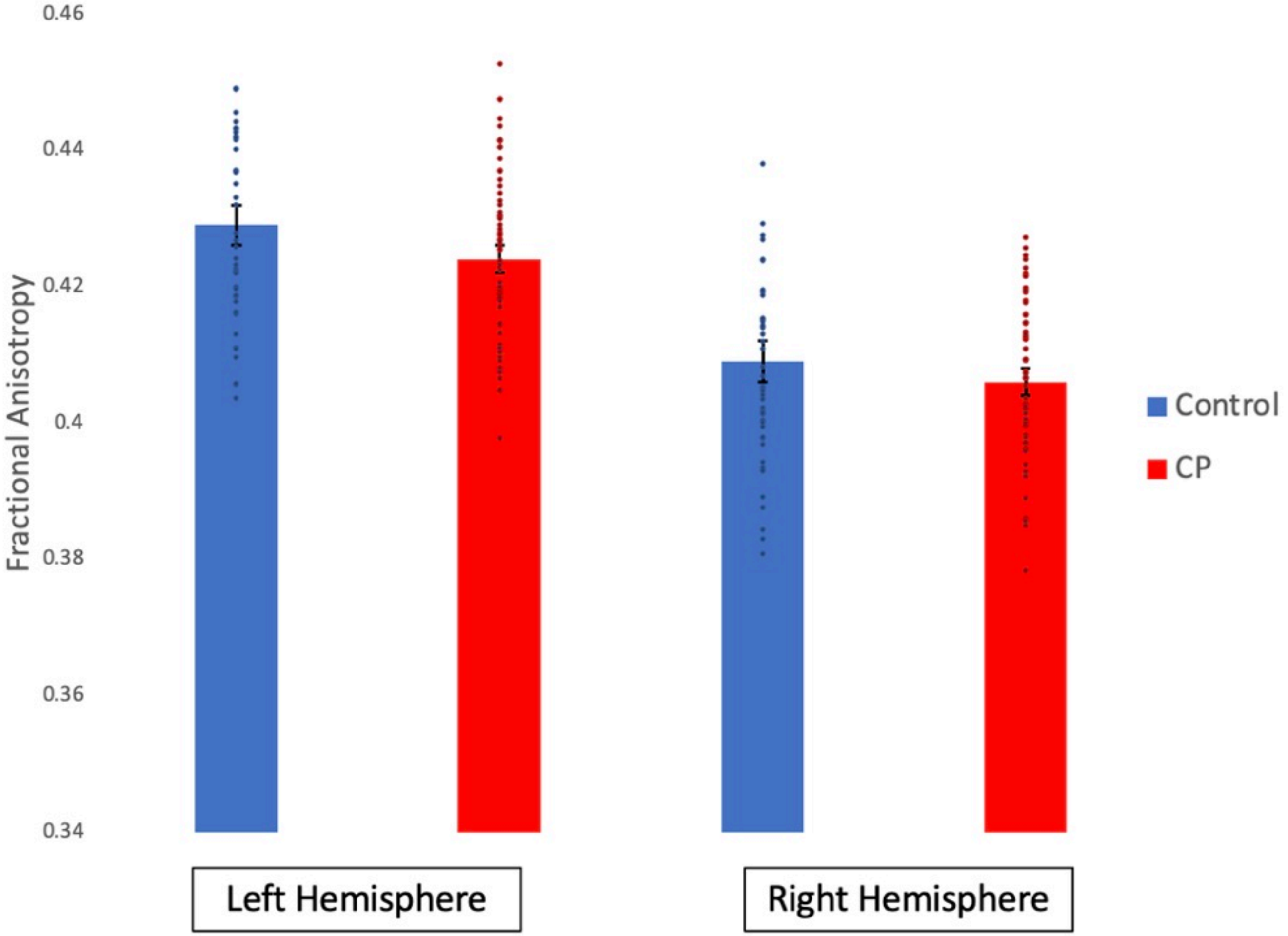

Hemisphere*Group *p*=0.034

Post hoc pairwise comparisons:

- Compared to the control group, the CP group have a non-significant decrease in FA in the left hemisphere (*p*=0.213) and the right hemisphere (*p*=0.445)
- Both groups have a significant decrease in FA (both *p*<0.001) in the right hemisphere
