# Supplementary material for: Rapid white matter changes in children with conduct problems during a parenting intervention
Source: Transl Psychiatry. 2023 Nov 4;13:339. doi: 10.1038/s41398-023-02635-8 (PMC10625622; doi:10.1038/s41398-023-02635-8)
Supplement: Supplementary file 3 — Fig S2 [file 41398_2023_2635_MOESM3_ESM.pdf]

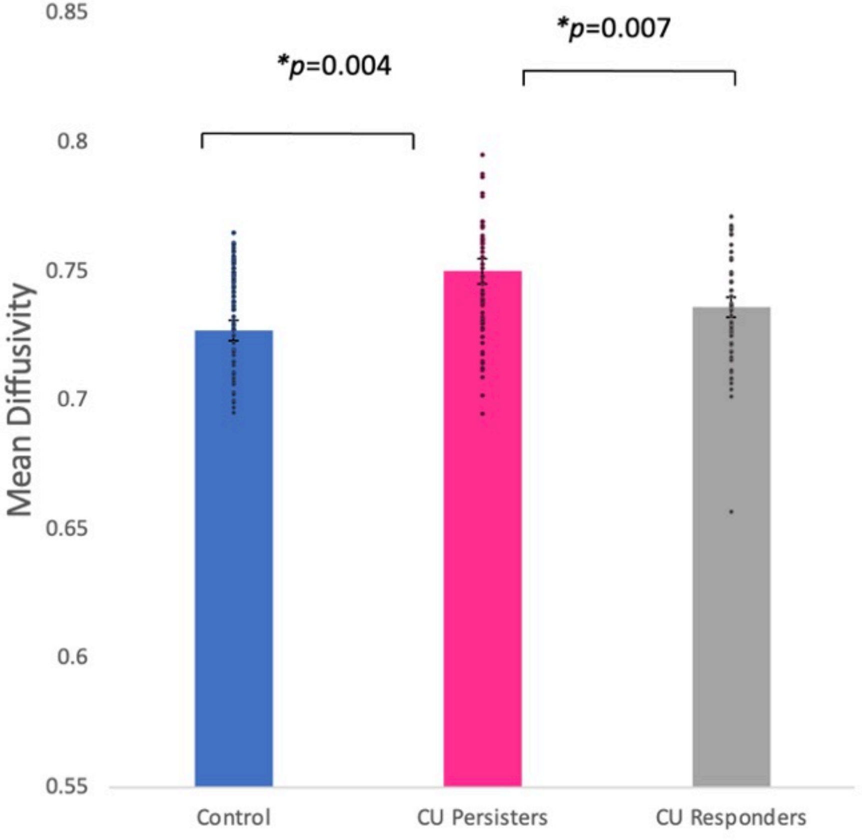

(Graph represents combined data across timepoints)

Significant main effect of RD (p=0.006)
**Post hoc* – see graph

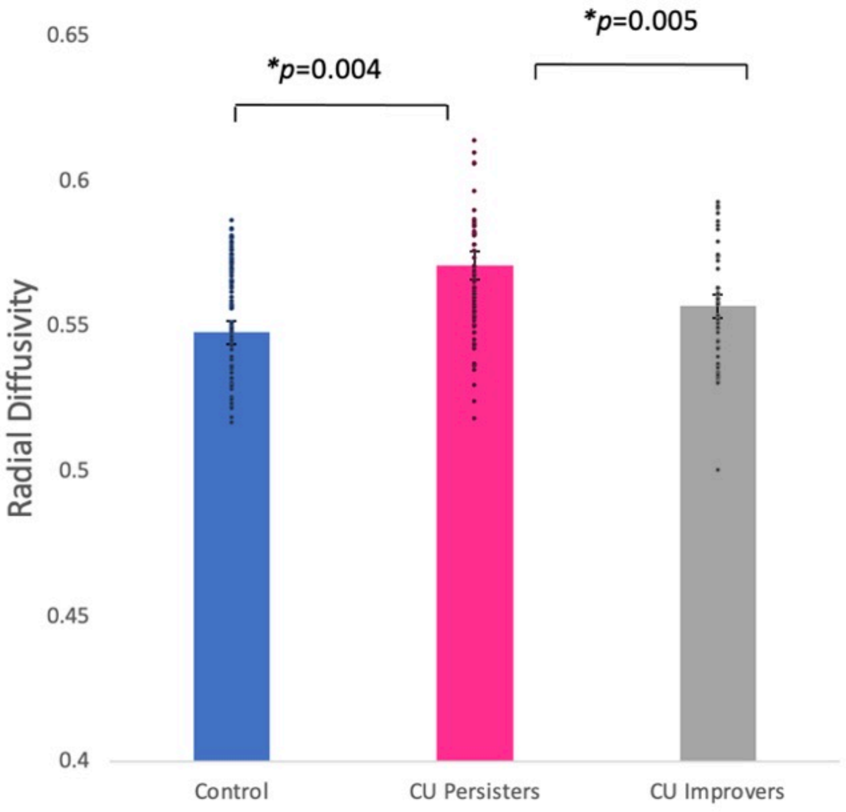

(Graph represents combined data across timepoints)

Significant main effect of MD (p=0.006)
**Post hoc* – see graph
